# Mapping of Leaf Rust Resistance Genes and Molecular Characterization of the 2NS/2AS Translocation in the Wheat Cultivar Jagger

**DOI:** 10.1534/g3.118.200058

**Published:** 2018-04-19

**Authors:** Shulin Xue, James A. Kolmer, Shuwen Wang, Liuling Yan

**Affiliations:** * Department of Plant and Soil Sciences, Oklahoma State University, Stillwater, OK 74078; † The Applied Plant Genomics Laboratory, National Key Laboratory of Crop Genetics and Germplasm Enhancement, Nanjing Agricultural University, Nanjing 210095, Jiangsu, China; ‡ United States Department of Agriculture, Agricultural Research Service, Cereal Disease Laboratory, University of Minnesota, St. Paul, MN 55108; § The Land Institute, KS 67401

**Keywords:** leaf rust, *Lr37*, *Lr17*, quantitative trait loci (QTL), wheat

## Abstract

Winter wheat cultivar ‘Jagger’ was recently found to have an alien chromosomal segment 2NS that has *Lr37*, a gene conferring resistance against leaf rust caused by *Puccinia triticina*. The objective of this study was to map and characterize the gene(s) for seedling leaf rust resistance in Jagger. The recombinant inbred line (RIL) population of Jagger × ‘2174’ was inoculated with leaf rust pathogen THBJG and BBBDB, and evaluated for infection type (IT) response. A major quantitative trait locus (QTL) for THBJG and BBBDB was coincidently mapped to chromosome arm 2AS, and the QTL accounted for 56.6–66.2% of total phenotypic variation in infection type (IT) response to THBJG, and 72.1–86.9% to BBBDB. The causal gene for resistance to these rust races was mapped to the 2NS segment in Jagger. The 2NS segment was located in a region of approximately 27.8 Mb starting from the telomere of chromosome arm 2AS, based on the sequences of the A genome in tetraploid wheat. The *Lr17a* gene on chromosome arm 2AS was delimited to 3.1 Mb in the genomic region, which was orthologous to the 2NS segment. Therefore, the *Lr37* gene in the 2NS segment can be pyramided with other effective resistance genes, rather than *Lr17a* in wheat, to improve resistance to rust diseases.

The hard red winter wheat variety Jagger (KS84W85/Stephens) (*Triticum aestivum*, 2*n* = 6×=42, AABBDD) has been widely grown in the Great Plains region of the U.S. since its release in 1996. Jagger was highly resistant to leaf rust caused by *Puccinia triticina* Erikss. when initially released, however within a few years races of *P. triticina* with virulence to *Lr17a* increased rapidly and the resistance in Jagger was much less effective (Long *et al.* 1998). Jagger is currently still used extensively as a parent due to the presence of many elite resistance genes for abiotic and biotic stresses, and many current hard red winter wheat cultivars in the U.S. have Jagger in their pedigrees (Fang *et al.* 2011; Liu *et al.* 2015, 2016; Turner *et al.* 2016).

Jagger was initially hypothesized to have leaf rust resistance gene *Lr17a*, based on its high similarity with the Thatcher near-isogenic line with *Lr17a* for infection types to different leaf rust races (Zhang *et al.* 2008; Kolmer 2017). Jagger was later determined to have the 2NS/2AS alien translocation (Fang *et al.* 2011) derived from *Aegilops ventricosa* Tausch (2*n* = 4×=28, DDNN) (Maia 1967) that was originally transferred to the wheat line VPM1 (Bariana and McIntosh 1993; Dyck and Lukow 1988). The 2NS/2AS translocation in VPM1 carries *Lr37* for leaf rust resistance, *Sr3*8 for resistance to stem rust caused by *P. graminis tritici* Eriks. & Henn., and *Yr17* for resistance to stripe rust, *P. striiformis* Westend. (McIntosh *et al.* 1995). A recombinant inbred line (RIL) population of Jagger × 2174 (IL71-5662/PL145) was tested for stripe rust resistance in different field locations, and the population segregated for *Yr17* and effective resistance to common stripe rust pathogen in Washington, Kansas and China (Fang *et al.* 2011). Jagger also has *Sr38* on the 2NS segment, in addition to *Sr7a* that conditions resistance to stem rust race TTTTF, which is the most virulent race found in North America and and is virulent to many winter wheat cultivars grown in the southern Great Plains (Turner *et al.* 2016). However, the effectiveness of the resistance genes on the 2NS segment in Jagger is not well known.

Leaf rust resistance conditioned by *Lr37* is best expressed in adult plants (McIntosh *et al.* 2016), however seedlings of the Thatcher line with *Lr37* have infection types (IT) that are usually mesothetic, with a mixture of medium to large uredinia with varying amounts of necrosis and chlorosis to avirulent races (Kolmer 1997). In a recent study Kolmer (2017) determined that seedling leaf rust resistance in the hard red wheat cultivar Santa Fe (G1878/Jagger) was conditioned by *Lr37* on the 2NS translocation.

The objectives of this study were to use the Jagger × 2174 RIL population to map the seedling leaf rust resistance genes in Jagger wheat, and to characterize the 2NS translocation with additional molecular markers. The Jagger × 2174 RIL population was recently genotyped with the Infinium iSelect 90K wheat bead chip array described in Cavanagh *et al.* (2013), which has facilitated discovery of additional resistance genes. The common wheat genome sequences recently released by the International Wheat Genome Sequencing Consortium (IWGSC) (https://urgi.versailles.inra.fr/blast_iwgsc/blast.php) have helped to develop markers for the translocated 2NS region, and the assembled genome sequences of the A genome in tetraploid emmer wheat (Avni *et al.* 2017) have helped to physically locate the resistance genes in common wheat.

## Materials and Methods

### The mapping population and inoculation

The parental lines Jagger and 2174, 141 F_7_ RILs derived from the parental lines, and a near-isogenic line of Thatcher wheat with *Lr17a*, RL6008 were phenotyped with leaf rust races THBJG and BBBDB in 2012 and 2017. The RIL population was arranged in six trays of 24 lines each, and a seventh tray of the remaining RIL and control lines. All entries were arranged as a randomized design. Each line was evaluated for reaction to leaf rust races. These materials were inoculated in the seedling stage with leaf rust races THBJG and BBBDB (Long and Kolmer 1989). Both races are avirulent to seedling plants with *Lr17a* and adult plants with *Lr37* (Helguera *et al.* 2003; Kolmer *et al.* 2005, 2006). Race THBJG is virulent to *Lr1*, *Lr2a*, *Lr2c*, *Lr3*, *Lr16*, *Lr26*, *Lr10*, *Lr14a*, and *Lr28*. In contrast, race BBBDB is virulent only to *Lr14a* and can be used to detect many seedling leaf rust resistance genes. Inoculation was conducted as previously described (Kolmer 2017). The parental lines and the RILs were grown in segmented trays with 8-10 seed of a single wheat line in each segment in a greenhouse at 20-23° and fertilized with 20-20-20 NPK solution 7 days after planting. At the two-leaf stage, the seedlings were inoculated with a single race of *P. triticina* using a suspension of urediniospores in Soltrol 170 (Phillips Petroleum, Bartlesville, OK) mineral oil. After inoculation the plants were dried for 1 hr, and then placed in a dew chamber overnight at 18°. The plants were then returned to a greenhouse bench at 20-23° with supplemental metal halide lighting. Leaf rust infection types were evaluated 10 days after inoculation.

### Infection type scores

Infection types (IT) of seedlings were scored as described by Long and Kolmer (1989) on a 0 to 4 scale: 0 = no visible sign of infection; ; = hypersensitive flecks; 1 = small uredinia with necrosis, 2 = moderate size pustules with chlorosis; 3 = moderate-large size uredinia without necrosis or chlorosis; 4 = large uredinia lacking necrosis or chlorosis. Mixtures of infection types were described with the lowest infection type first. Symbols “–” and “+” denote smaller or larger uredinia. The original phenotypic data were converted to a 0–9 linear disease scale for QTL analysis. IT ;, ;1^-^, ;1, ;1^+^, ;2^-^, 2, 2^+^, 3^-^, 3, and 3^+^ were coded as 0, 1, 2, 3, 4, 5, 6, 7, 8 and 9, respectively. For plants in the same RIL that showed heterogeneous infection types, only the most prevalent IT was used.

### Genotypes

The 90K Illumina wheat SNP chip was used to genotype the Jagger × 2174 population of 141 RILs (Li *et al.* 2015), and an 8-digit SNP code for the mapping population was converted to the reference number for each SNP, *i.e.*, IWA0000 or IWB0000 as published (Cavanagh *et al.* 2013). However, the sequences of three SNP markers, SNP19777336, SNP30671452 and SNP48765306, were not found to have a reference number, and their sequences for mapping in this study were provided in supplementary information. The SNP markers mapped to chromosome 2A were incorporated with SSR markers and gene markers reported in previous studies (Fang *et al.* 2011; Tan *et al.* 2013). Those lines that contained multiple crossovers within a small chromosomal region were excluded for further analyses. Final genotypic data and phenotypic data were integrated in 129 RILs to analyze QTL. QTL were found using MapQTL 6.0. Only those SNP markers that covered a significant QTL were used to further analyze genetic distance using MapMaker 3.0, with the Kosambi mapping function to estimate the map distance. WinQTLCart 2.5 (North Carolina State University, Raleigh) was used to conduct analyses using Composite Interval Mapping (CIM), and a QTL was declared when the logarithm of the odds (LOD) score exceeded the LOD threshold that was determined at 300 permutations and significance level at 0.05.

### Development of sequence tagged site (STS) markers

PCR markers for three genes, *TaVrga-A1* and *cMWG682* containing fragments encoding conserved domains of resistance genes (Fang *et al.* 2011), and *TaOPR2* for a 12-oxo-phytodienoic acid reductase (*OPR*) gene (Tan *et al.* 2013), were mapped to the 2NS segment in Jagger. To verify the genotype of the major QTL for IT response to leaf rust races THBJG and BBBDB, 13 STS markers specific to chromosome 2A were developed, based on seven SNP markers, two SSR markers, and five putative genes. The primers for these STS markers and the expected sizes of PCR products are provided in Table 1.

**Table 1:** Primer sequences of the markers used in this study

Markers	Forward primer (5′-3′)	Reverse primer (5′-3′)	PCR (bp)	Tm (°C)
Traes_2AS_3381A39C6	GTTCCACACCCAAGATGGTTAC	TGAAAGAAACACTAAGCTGAGGAG	503	55
SNP19777336	TGAATATATAGCAGTATGGGTGGCAG	GTACCAAACGCCATGAAAGGG	1075	55
IWB58709	GGTTGAGTTTCCGAGTTTCGTG	GTTCTTGTTCTCCCGTTTGTGC	1505	58
GWM636-M	TGCGGTAGTTTTTAGCAAAGCG	TTTGTCTGGATAATACGACCCCC	738	56
IWB64569	AGCCCTTGCGGAATTCATGGCAAA	GCTACAGCAGTATGTACACAAAAGCCTG	1351	55
Traes_2AS_5CAF7A367	TCCATCCACAACACCTACCC	GCAGCGTAATAATACTTCCTATGAT	1725	55
IWB72490	CTCCACCGCTCATCTTCCTG	TCCCCCAACCGACATTATCC	1559	55
WMC407-M	GCATCTTGTGTTGGTGTTGCC	TCAAGGGTGCGTTTTTCACTTC	423	53
Traes_2AS_D11AD107B	GTTCGCATCTCCGCCTTTAGG	TGGTCACAGGGTAGAAGTATTTCGG	1617	55
Traes_2AS_27D2CB02A	CAAGACACACAGACCTGCTTTTT	CACTGTAATAAAGTGGTAAAGTGAAGC	1675	53
IWB11136	GTCTACATGAAAATAAGCTGCTGAGG	GATGGAGTTTTGGGTTTAAGTTCTAG	365	52
SNP30671452	TGGAGGAAGACGATGGAGACTG	GCAAACCACCAAAGAAGAACCG	581	61
IWB47995	GCTCCCCAAGGACAACATCA	AGAGAGCCAAGAGCAAGCATACC	497	60

Seven SNP markers and two SSR markers (*Xgwm636* and *Xwmc407*) were converted to 2A-specific PCR markers based on the 100 bp SNP probe sequences and the genome sequence harboring the target SSR. The sequences were used as queries to search the IWGSC Chinese Spring wheat scaffold sequences of 2A, 2B and 2D (https://urgi.versailles.inra.fr/blast_iwgsc/blast.php). The BLASTN search was limited to the top hit with an E-value cut off of at least 1E-10. The resulting scaffolds were used to design primers that were specific to chromosome 2A to amplify the fragment covering the SNP or SSR marker sequence region. The amplified PCR products were confirmed to be on chromosome 2A by sequencing PCR products. Five putative *T. aestivum* genes residing within the QTL region, *Traes_2AS_3381A39C6* encoding a hypothetical protein, *Traes_2AS_5CAF7A367* encoding a NB-ARC domain-containing disease resistance protein, *Traes_2AS_67EFE0FAE* encoding a heat shock protein 90 (HSP90), *Traes_2AS_D11AD107B* encoding a hypothetical protein, and *Traes_2AS_27D2CB02A* encoding a mortality factor 4-like protein 1, were chosen as representatives to distinguish between the 2NS segment and the orthologous region of chromosome 2A in hexaploid wheat.

The PCR was performed by using a *Taq* DNA polymerase (New England BioLabs) and 35 thermal cycles after denaturing at 95° for 3 min, with each cycle consisting of 94° for 30 sec, 55-61° for 40 sec, and 72° for 1-2 min, and a final extension at 72° for 10 min. The annealing temperature and extension time of PCRs were determined depending on primers and product size. All of the developed STS markers but IWB47995 showed a dominant pattern for chromosome arm 2AS with presence in 2174 but absence in Jagger. The STS marker for IWB47995 is co-dominant, and the 497 bp fragment was digested with restriction enzyme *Hpa* II that produced 328 bp and 169 bp fragments for the allele from Jagger and 345 bp and 152 bp fragments for the allele from 2174.

### Physical mapping and sequence annotation

To determine the physical position of markers on chromosome arm 2AS, the sequences corresponding to the markers were used as BLASTN queries against the assembled pseudomolecules of chromosome 2A (https://urgi.versailles.inra.fr/download/iwgsc/IWGSC-WGA/) with an E-value cut off of at least 1E-10. The physical distance between *Xgwm636-M* and *Xwmc407-M* was predicted based on the assembled genome sequences of the A genome in tetraploid emmer wheat (http://z-data.interomics.eu) (Avni *et al.* 2017). The genes residing in the region between *Xgwm636-M* and *Xwmc407-M* on chromosome 2A were predicted according to the IWGSC Survey sequence annotations (https://wheat-urgi.versailles.inra.fr/Seq-Repository/Genes-annotations). Their putative functions were determined, via BLASTX searches, against the National Centre for Biotechnology Information (NCBI) non-redundant protein sequence database using an E-value cut-off of 1E-5.

### Data availability

The authors state that all data necessary for confirming the conclusions presented in the article are represented fully within the article. Supplemental material available at Figshare: https://doi.org/10.25387/g3.6122270.

## Results

### Infection type response of the Jagger × 2174 RIL population to THBJG and BBBDB

Seedlings of Jagger had low IT (;1^-^) to THBJG, but seedlings of 2174 had high IT (3^+^) to THBJG (Table 2), indicating that race THBJG can be used to detect genes in the Jagger × 2174 RIL population. Both Jagger (;1^-^) and 2174 (;) had low IT to BBBDB. The control line RL6008 carrying the *Lr17a* gene showed the same IT (;1^-^) as Jagger to both THBJG and BBBDB. The Thatcher line (RL6081) with *Lr37* on the 2NS translocation had IT of:22^-^ to BBBDB, and 2^+^3 to THBJG. The lower IT of Jagger indicated that Jagger has additional resistance to both races compared to RL6081.

**Table 2:** IT response to leaf rust races THBJG and BBBDB in parental lines, controls and RILs

Race	Parents		Controls		Population	
	Jagger	2174	RL6008-*Lr17a*	RL6081-*Lr37*	Mean	Min–Max
THBJG	1*^a^* (;1^-^)*^b^*	9 (3^+^)	1 (;1^-^)	6.5 (2^+^3)	6.2	0–9
BBBDB	1 (;1^-^)	0 (;)	1 (;1^-^)	(5.5) ;22^-^	4.7	0–9

a converted IT used for QTL analysis.

b original IT.

**Figure 1:**
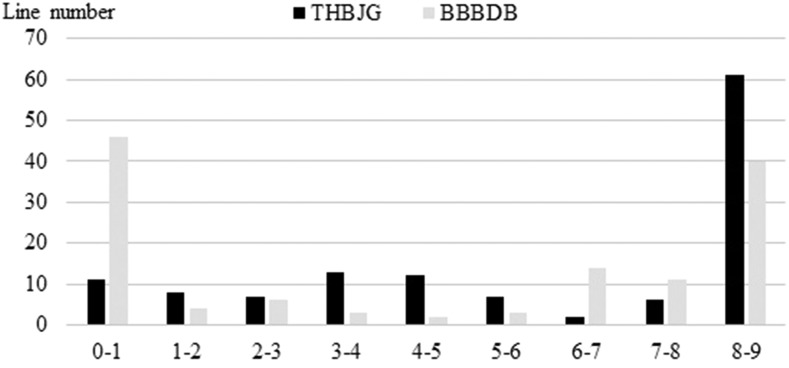
Distribution of the Jagger × 2174 RILs infection type to THBJG and BBBDB. Frequency distribution of the Jagger × 2174 RILs for converted IT using the 0–9 linear disease scale. Y axis indicates the number of lines with different ITs in the population of 129 RILs. The converted ITs were averaged from two tests in 2012 and 2017.

The ITs of 129 Jagger × 2174 RILs inoculated with race THBJG and race BBBDB individually as seedlings in 2012 and 2017 were analyzed in this study. The correlation of IT between the two tests was highly significant for THBJG (r = 0.829, *P* < 0.001) and for BBBDB (r = 0.829, *P* < 0.001). The average converted IT of the two tests for the whole population was 6.2 for THBJG, whereas the average converted IT for both tests was 4.7 for BBBDB (Table 2), indicating THBJG had higher virulence than BBBDB to the Jagger × 2174 population. To race BBBDB 60 RILs had IT of 3^+^ (at 9 in scale) in both tests, 45.7% of the total RILs. To race THBJG 59 RILs had IT of 3^+^ in both tests, 46.5% of the total RILs. These results suggested that the segregation of the 129 RILs to both races fit a single major gene ratio of 1:1 (*P* > 0.05). The converted ITs of the RILs to both races ranged from 0 to 9 (Figure 1), The RILs with the lowest ITs likely have some additional minor genes with small effect on IT, in addition to the single major resistance gene that segregated.

### A major QTL for resistance to THBJG and BBBDB was mapped to the 2NS segment

**Figure 2:**
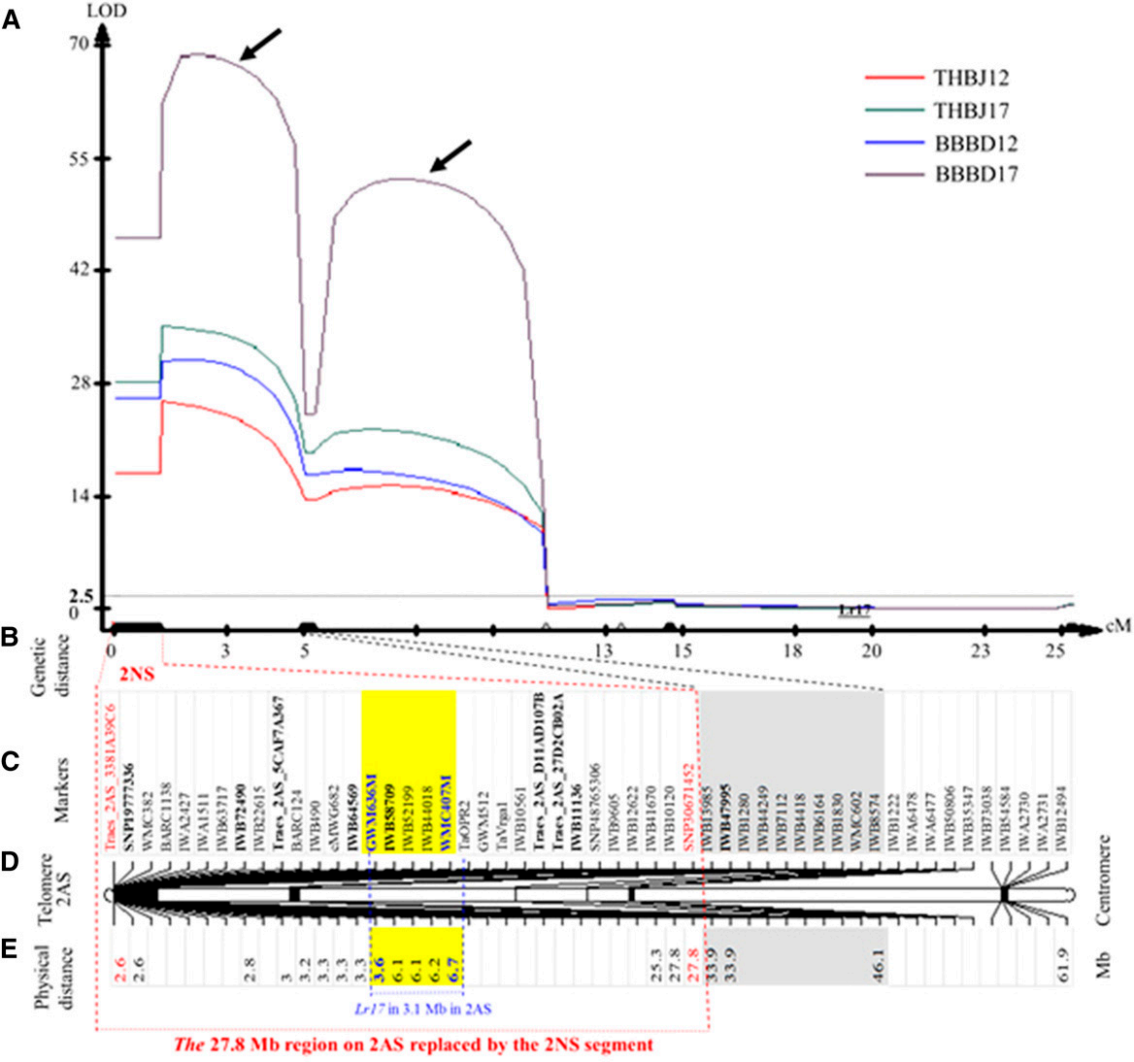
Genetic and physical relationship between *Lr17a* and the 2NS segment containing *Lr37*. A). A major QTL for reaction to THBJG and BBBDB. The genetic map was constructed using MapMaker 3.0 and QTL graphs were constructed using WinQTL Cart 2.5. The vertical dotted line indicates the logarithm of the odds (LOD) significance threshold of 2.5. Arrows point to peaks, under which no marker was mapped, and the peaks should be false. B). Genetic distance of the QTL for resistance to THBJG and BBBDB. C). Markers in the linkage group spanning the QTL. Markers that were converted to STS markers for mapping of the QTL are shown in bold. Markers that were used to determine the physical distance spanning the QTL are shown in red. The markers on chromosome arm 2AS that showed recombination with the 2NS segment are highlighted with gray shaded box. D). A diagram of genetic locations of the mapped markers on chromosome arm 2AS. E). Physical locations of the 3.1 Mb region including *Lr17a* on chromosome arm 2AS. Markers that were used to determine the physical distance of the *Lr17a* region are shown in yellow.

A major QTL for reaction to THBJG and BBBDB was coincidently mapped to the 2NS region (Figure 2A). The LOD value at the peak position of the QTL for THBJG was 25.9 and 35.4, and accounted for 56.5% and 62.2% of total phenotypic variation, in 2012 and 2017 respectively. The LOD value at the peak position of the QTL for BBBDB was 31.1 and 69.1, and accounted for 72.2% and 86.9% of total phenotypic variation, in 2012 and 2017 respectively.

A total of 52 markers was assembled to a linkage group spanning approximately 26 cM, with a marker density of 0.53 cM per marker (Figure 2B). The linkage group included 38 SNP markers, seven SSR markers, and seven gene markers (Figure 2C). The peak of the QTL was covered by a cluster of 32 markers from Traes_2AS_3381A39C6 closest to the telomere (Figure 2D), throughout the five markers *Xwmc382*, *Xbarc1138*, *Xbarc124*, *Xgwm512* and *TaVrga1*, which were previously mapped to 2NS (Fang *et al.* 2011), to SNP30671452 which is the 33^rd^ marker in the linkage group. These clustered markers showed no crossover (Table S1).

### The physical distance of the 2A chromosomal region replaced by the 2NS segment

Sequences of the SNP and STS markers were used to search the recently released the emmer wheat genomic sequences, which allowed a physical distance covering the clustered markers on chromosome 2A to be determined. The SNP markers were identified in scaffolds of chromosome 2A (Table S1). On the side closest to the telomere, the first clustered marker for the QTL peak was *Traes_2AS_3381A39C6* located at position 2.6 Mb of chromosome arm 2AS (Figure 2E), and the last marker in the cluster was *SNP30671452* which was at position 27.8 Mb of chromosome arm 2AS; therefore, the clustered markers for the QTL peak were predicted to cover 27.8 Mb regardless of the distance to the centromere.

**Figure 3:**
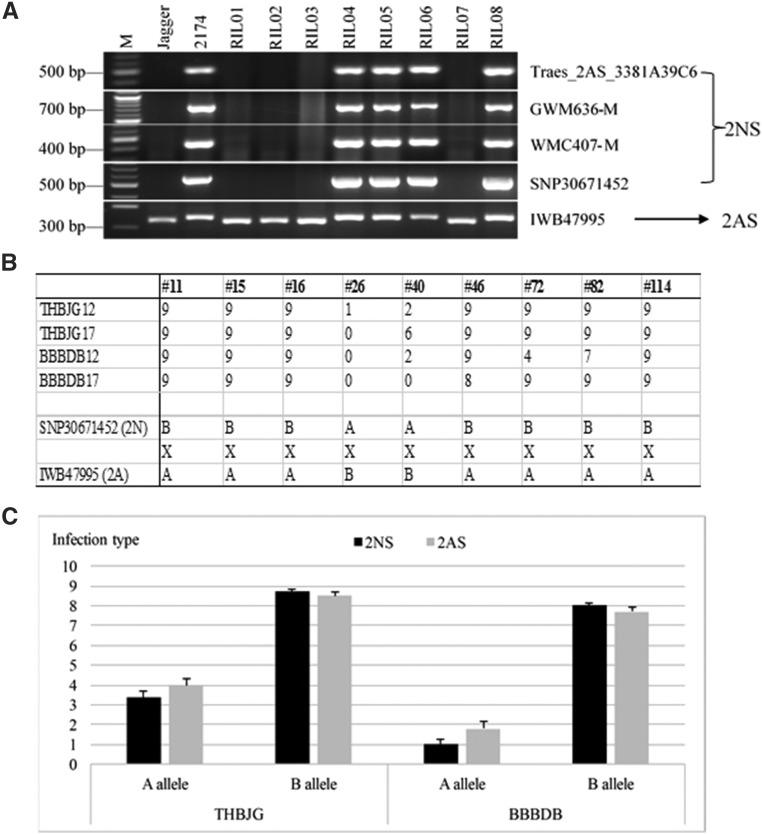
Development and utilization of STS markers for the 2NS segment. A). Mapping of five STS markers in eight random lines. M: 100-bp ladder. The numbers to the left indicate the molecular size in bp. The first four markers showed the presence/absence polymorphism, and the last marker IWB47995 shows the polymorphic band pattern after the PCR products were digested with *Hpa* II. B). Genotypes and phenotypes of nine critical recombinant lines. ‘A’ represents the Jagger allele, ‘B’ represents the 2174 allele, and ‘x’ indicates a crossover between two markers. The phenotype is infection type scored in the 0-9 scale. C). Genetic effects of the 2NS segment to THBJG and BBBDB. The converted ITs were averaged from two tests in 2012 and 2017, and bar indicates standard error (± SE). ‘A’ indicates the Jagger allele in the 2NS region including SNP30671452 and the 2AS region including IWB47995, and ‘B’ indicates the 2174 allele in the 2NS region and the 2AS region.

The 2A-specific primers for 13 markers included in the cluster (Table 1) were confirmed to amplify PCR products from chromosome 2A but failed to amplify any products from the 2NS segment, and all of these markers had dominant inheritance, showing their absence was associated with the A allele in Jagger and their presence was associated with the B allele in 2174. Four of the STS markers for 2NS/2AS are shown in Figure 3A.

On the 2AS segment closest to the 2NS segment, the SNP marker IWB47995 which showed recombination with the clustered markers for the 2NS segment, was converted to a cleaved amplified polymorphic sequence (CAPS) marker. In the sequenced 497 bp fragment, three nucleotides were found to differ between Jagger and 2174 alleles, and two of the SNPs were located within the recognition site of restriction enzyme *Hpa* II. The CAPS marker for IWB47995 was mapped as a co-dominant polymorphism between the two alleles (Figure 3A). IWB47995 was assigned to position 33.9 Mb from the telomere of chromosome arm 2AS in emmer wheat (Figure 2E).

Jagger had the same sequence in IWB47995 as Chinese Spring; therefore, the genome region from IWB47995 to the centromere in Jagger should be chromosome arm 2AS from hexaploid wheat (Figure 2D). Since 50 SNP markers in the 27.8-33.9 Mb region of chromosome arm 2AS (Table S2) were monomorphic between the two alleles and these markers had identical sequences to Chinese Spring, the 27.8-33.9 Mb region in Jagger should also be chromosome arm 2AS. Therefore, the 2AS region replaced by the translocated 2NS segment was approximately 27.8 Mb (Figure 2E), according to the physical distance of assembled genomic sequences of chromosome 2A in emmer wheat.

Nine lines were found to have a crossover between SNP30671452 representing the 2NS segment and IWB47995 representing chromosome arm 2AS (Figure 3B). Among seven of these critical recombinant lines that carried the B allele from chromosome arm 2AS, all had highest converted IT to THBJG, and all of them had high or intermediate susceptibility to BBBDB (Figure 3B). Two critical recombinant lines (RIL26 and RIL40) that carried the A allele on the 2NS segment had very low or intermediate converted IT to THBJG and BBBDB. The tight linkage of 2NS with the resistance of the nine critical recombinant lines indicated that the seedling resistance genes detected in the Jagger × 2174 RILs was present in the 2NS segment.

At the population level as shown in Figure 3C, in the region of SNP30671452 representing the 2NS segment, the average converted IT of those lines carrying the A allele was 1 for BBBDB and 3.4 for THBJG, whereas the average IT of those lines carrying the B allele was 8 for BBBDB and 8.7 for THBJG. However, in the IWB47995 region representing chromosome arm 2AS, the average IT of those lines carrying the A allele was 1.8 for BBBDB and 4 for THBJG, whereas the average IT of those lines carrying the B allele was 7.7 for BBBDB and 8.7 for THBJG.

### The physical location of Lr17a on chromosome arm 2AS

The wheat *Lr17a* gene was mapped close to the distal end of the short arm of chromosome 2A, at 2.7-4.0 cM to *Xgwm636* and at 2.5 cM to *Xwmc407* (Bremenkamp-Barrett *et al.* 2008; Zhang *et al.* 2008). GWM636 and WMC407 were converted to 2A-specific STS markers (Table 1) that were mapped in the population of Jagger × 2174 RILs. The dominant band patterns of the modified PCR markers for the SSR markers demonstrated that they were linked with the 2NS segment (Figure 3A).

Sequences of GWM636-M and WMC407-M were used to search the emmer wheat genome sequence, and GWM636-M was found on scaffold33664 at position 3.6 Mb, and WMC407-M was found on scaffold98927 at position 6.7 Mb. Based on these results, the physical distance between *Xgwm636-M* and *Xwmc407-M* was at least 3.1 Mb (Figure 2E), according to the emmer wheat genome sequences.

## Discussion

Seedling leaf rust resistance in Jagger wheat mapped to the 2NS translocation derived from *A. ventricosa*. The 2NS translocation in Jagger was determined to span 27.8 Mb on the distal region of chromosome arm 2AS as delineated by molecular markers. Both races THBJG and BBBD produced a very low IT that mapped to the 2NS translocation. *Lr37* has been regarded as an adult plant resistance gene, since lines with the 2NS translocation optimally express resistance in adult plants, and low IT can be difficult to obtain in seedlings (McIntosh *et al.* 1995). However, wheat populations segregating for the 2NS translocation have also segregated for seedling leaf rust resistance (Dyck and Lukow 1988; Kolmer 2017). At normal greenhouse temperatures of 20°, seedlings of the Thatcher line with *Lr37* condition a mesothetic response of necrosis, chlorosis, and various sized uredinia (IT X^+^) (Kolmer 1997). Temperature also affects expression of *Lr37*. At 17° wheat lines with the 2NS translocation expressed low IT 12 compared to IT 3 at 20° (Bariana and McIntosh 1994).

Previously *Lr17a* was postulated to be present in Jagger (Zhang *et al.* 2008; Kolmer 2017) and many other hard red winter wheat cultivars in the southern Great Plains region of the U.S that were derived from Jagger. This was reinforced by the loss of resistance in Jagger and related cultivars due to the rapid increase of races with *Lr17a* virulence in North America after the release of Jagger in 1996. Our study placed the *Lr17a* locus on 2AS within a 3.1 Mb region, which is orthologous to the 2NS translocation, thus eliminating the possibility of *Lr17a* in Jagger. Bariana and McIntosh (1993) previously determined that the 2NS translocation and *Lr17a* were in linkage repulsion since they did not recover any F_2_ plants with both in a cross between a wheat line with the 2NS translocation and the Thatcher line with *Lr17a*. The *P. triticina* races with virulence to *Lr17a* that have been prevalent in North America since the mid 1990s are also virulent to *Lr37* (Kolmer *et al.* 2005). The *Lr17 locus has* two resistance alleles, *Lr17a originally found in* wheat cultivars Rafaela and EAP 26127 (Dyck and Kerber 1977) and *Lr17b* in the Australian cultivar Harrier (Singh *et al.* 2001). *Lr17a* and *Lr17b* alleles also have similar IT to the same races, depending on the temperature and leaf rust race (Singh *et al.* 2001). Since *Lr17a*, *Lr17b* and *Lr37* are found in the same region on 2AS and 2NS respectively, these genes may be allelic at the same corresponding loci in the different genomes. Genes *Lr2a*, *Lr2b*, and *Lr2c* are allelic and are highly related for IT to avirulent leaf rust pathogen (Dyck and Samborski 1974). Since the 2NS translocation and the *Lr17* locus are linked in repulsion it is not possible to conduct classical allelism tests. Further sequencing and characterization of the corresponding regions on 2NS and 2AS may determine the relationship between *Lr37* and the *Lr17* genes. In addition, Jagger carrying *Lr37* on 2NS can be crossed with a cultivar/line that carries *Lr17* on 2AS. This population would segregate for both *Lr37* and *Lr17*, therefore, the two genes could be compared for resistance in the same genetic background.

The wheat *Lr17* gene was physically delimited into the 3.1 Mb region between *Xgwm636-M* and *Xwmc407-M*. All of the resistance genes residing in the targeted region on chromosome arm 2AS should be candidates for *Lr17a* in wheat. Four genes were predicted to be related with known resistance genes in the 3.1 Mb region between *Xgwm636-M* and *Xwmc407-M*, including *Traes_2AS_5CAF7A367* (encoding a NB-ARC domain-containing disease resistance protein), *Traes_2AS_089875521* (encoding a disease resistance RPP13-like protein 1), *Traes_2AS_6E1B0E6EF* (encoding a resistance gene analog 2 (RGA2), and *Traes_2AS_AFEB22E37* (encoding a disease resistance RPP13-like protein 1). In addition, another two genes are located in this region, including *Traes_2AS_D0C21ADB5* that was predicted to encode a WRKY transcription factor, and *Traes_2AS_7B74BE6D6* that was predicted to encode a putative leucine-rich-repeat (LRR) receptor-like serine/threonine-protein kinase. Further study is needed to test the functions of these candidate genes in wheat.

An unresolved issue in this study was that the resistance to BBBD in 2174 was not mapped. Recombinant inbred lines with resistance alleles to BBBD derived from 2174 were not identified. 2174 was also resistant to stripe rust when the same population was tested in China (Fang *et al.* 2011), but this resistance was also not mapped. Although the Jagger × 2174 RIL population has been genotyped using the wheat 90K SNP assay, not all genomic regions may have been covered, and the uncharacterized part of the genome may have additional stripe and leaf rust resistance genes.

In summary, the seedling resistance in Jagger was mapped to the 2NS translocation. The 2NS translocation in Jagger was determined to be 24 Mb in length, on the distal region of 2A. The leaf rust resistance gene locus *Lr17* on 2AS, mapped in a 3.1 Mb region, which is orthologous to the 2NS translocation. *Lr37* on the 2NS translocation may be allelic with the *Lr17* locus on 2AS. The population in North America has high level of virulence to *Lr17a* and *Lr37*, however the three minor QTL may be useful in combinations with other effective resistance genes in development of leaf rust resistance cultivars.
